# Skeletal Muscle Tissue Saturation Changes Measured Using Near Infrared Spectroscopy During Exercise Are Associated With Post-Occlusive Reactive Hyperaemia

**DOI:** 10.3389/fphys.2022.919754

**Published:** 2022-07-08

**Authors:** Siana Jones, Therese Tillin, Suzanne Williams, Alicja Rapala, Nishi Chaturvedi, Alun D. Hughes

**Affiliations:** MRC Unit for Lifelong Health & Ageing at UCL, Department of Population Science and Experimental Medicine, Institute for Cardiovascular Science, University College London, London, United Kingdom

**Keywords:** near-infrared spectroscopy, skeletal muscle, reactive hyperaemia, exercise, microvascular

## Abstract

Measuring local haemodynamics in skeletal muscle has the potential to provide valuable insight into the oxygen delivery to tissue, especially during high demand situations such as exercise. The aim of this study was to compare the skeletal muscle microvascular response during post-occlusive reactive hyperaemia (PORH) with the response to exercise, each measured using near-infrared spectroscopy (NIRS) and to establish if associations exist between muscle measures and exercise capacity or sex. Participants were from a population-based cohort study, the Southall and Brent Revisited (SABRE) study. Skeletal muscle measures included changes in tissue saturation index at the onset of exercise (∆TSI_BL-INC_) and across the whole of exercise (∆TSI_BL-EE_), time to 50%, 95% and 100% PORH, rate of PORH recovery, area under the curve (AUC) and total oxygenated Haemoglobin (oxy-Hb) change during PORH. Exercise capacity was measured using a 6-min stepper test (6MST). Analysis was by multiple linear regression. In total, 558 participants completed the 6MST with NIRS measures of TSI (mean age±SD: 73 ± 7years, 59% male). A sub-set of 149 participants also undertook the arterial occlusion. Time to 100% PORH, recovery rate, AUC and ∆oxy-Hb were all associated with ∆TSI_BL-EE_ (β-coefficient (95%CI): 0.05 (0.01, 0.09), *p* = 0.012; −47 (-85, −9.9), *p* = 0.014; 1.7 (0.62, 2.8), *p* = 0.002; 0.04 (0.002.0.108), *p* = 0.041, respectively). Time to 95% & 100% PORH, AUC and ∆oxy-Hb were all associated with ∆TSI_BL-INC_ (β-coefficient (95%CI): −0.07 (−0.12,−0.02), *p* = 0.02; −0.03 (−0.05, −0.003), *p* = 0.028; 0.85 (0.18, 1.5), *p* = 0.013 & 0.05 (0.02, 0.09), *p* = 0.001, respectively). AUC and ∆Oxy-Hb were associated with steps achieved (β-coefficient (95%CI): 18.0 (2.3, 33.7), *p* = 0.025; 0.86 (0.10, 1.6), *p* = 0.027). ∆TSI_BL-EE_ was associated with steps and highest VO_2_ (1.7 (0.49, 2.9), *p* = 0.006; 7.7 (3.2, 12.3), *p* = 0.001). ∆TSI_BL-INC_ was associated with steps and VO_2_ but this difference was attenuated towards the null after adjustment for age, sex and ethnicity. ∆TSI_BL-EE_ was greater in women (3.4 (0.4, 8.9) versus 2.1 (0.3, 7.4), *p* = 0.017) and ∆TSI_BL-INC_ was lower in women versus men (2.4 (0.2, 10.2) versus 3.2 (0.2, 18.2), *p* = 0.016). These Local microvascular NIRS-measures are associated with exercise capacity in older adults and several measures can detect differences in microvascular reactivity between a community-based sample of men and women.

## Background

Reduced aerobic exercise capacity is a risk factor for future CVD morbidity and mortality. (Laukkanen et al., 2007; Kodama et al., 2009; Hamer et al., 2020). As adequate supply of oxygenated blood to skeletal muscle is one of the primary components of aerobic exercise capacity, measuring local haemodynamics in skeletal muscle during exercise has the potential to provide valuable insight into the oxygen transport pathway. However, assessment of skeletal muscle haemodynamics during exercise, particularly at the microvascular level, is challenging.

Near-infrared Spectroscopy (NIRS) offers a simple non-invasive technique that measures changes in oxygenated and deoxygenated haemoglobin and myoglobin (oxy-Hb & deoxy-Hb) and tissue saturation index (TSI) specifically from small blood vessels and myocytes. (Scholkmann et al., 2014). Development of NIRS technology into small, wireless devices permits measurements to be captured during exercise. A number of reports suggest that greater drops in TSI during exercise represent local impairment in oxygen delivery (Boezeman et al., 2016) and there is good evidence for the use of TSI in clinical setting for assessment of peripheral vascular diseases. (Bauer et al., 2004; Mesquita et al., 2013). However, NIRS has not yet been adopted in routine clinical practice and evidence investigating its sensitivity to detect differences in non-clinical population-based samples, particularly in older adults, is limited. Despite recognised sex differences in the pattern of cardiovascular aging, (Redfield et al., 2005; Chung et al., 2006) including a differential pathophysiology in development of heart failure that involves microvascular dysfunction in women, (Beale et al., 2018) NIRS has been under-employed in investigating these differences. (Jones et al., 2021a).

NIRS can also be applied during and following a cuff-induced arterial occlusion, the subsequent post occlusive reactive hyperaemia (PORH) can be quantified to provide fairly well established indices of microvascular reactivity. (Rosenberry and Nelson, 2020). The PORH reperfusion slope is associated with post-occlusive brachial artery blood flow. (Bopp et al., 2014). Strong evidence suggests the reperfusion slope is slower and time to peak recovery longer in the presence of peripheral arterial disease (PAD). (Cheatle et al., 1991; Kooijman et al., 1997; Chung et al., 2018). Surprisingly, a comparison of the outcomes derived from application of NIRS to assess TSI during exercise and its application during PORH has not previously been carried out. Furthermore, the TSI increase at initiation of exercise (oxy-Hb over-shoot), which has the potential to provide a crude indicator of vasodilatory capacity at initiation of exercise, is not widely described and limited prior work examining an ‘overshoot’ in oxy- or deoxy-Hb has used measurements derived from single distance NIRS devices which are unable to spatially resolve the signals. (Bowen et al., 19852012; Salzmann et al., 2021).

Therefore, the objectives of this study are: 1) to compare changes in skeletal muscle tissue saturation index (TSI) during exercise with indices of microvascular function captured during PORH 2) to determine if NIRS-derived measures of microvascular function are associated with exercise capacity in older adults, and 3) to compare skeletal muscle microvascular function in men versus women in a population-based sample of older adults.

## Methods

### Participants

Participants were drawn from a population-based, tri-ethnic cohort study of older adults resident in West London, United Kingdom: the Southall and Brent Revisited (SABRE) study. (Tillin et al., 2012). Data presented in this study were collected at the 25–30 years follow-up visit (2015–2018). Participants were excluded from undertaking exercise tests according to co-morbidity contraindications given in the American College of Sports Medicine (ACSM) guidelines. (American College of Sports Medicine, 2018).

All procedures were in accordance with the principles of the Helsinki declaration, all participants gave written informed consent and the study was approved by the National Research Ethics Service (NRES) Committee London—North Fulham.

### Anthropometrics and Questionnaires

Height was measured barefoot using a stadiometer (Seca 217; Seca, Hamburg, Germany). Weight was measured using digital bio-impedance scales (BC-418; Tanita, IL, United States). Adipose tissue thickness (ATT) overlaying the NIRS measurement site was measured using an ultrasound device (Vivid I; GE, Boston, MA, United States) fitted with a high frequency transducer (12L-RS; 6–13 MHz; GE).

Information on physical activity, smoking, history of cardiovascular disease (CVD), hypertension and medication use were obtained by questionnaire. Diabetes was defined as self-reported physician diagnosis, reported use of glucose-lowering medication or an elevated measurement of HbA_1c_ above the guideline cut-off value for diagnosis of type 2 diabetes (≥48 mmol/mol [>6.5%]) (Chung et al., 2018). HbA_1c_ was measured using an immunoassay (cobas HbA_1c_ test) on the Cobas c311 automated analyser (Roche Diagnostics, Burgess Hill, United Kingdom). Non-fasting blood samples were obtained in the morning of the clinic visit.

### Aerobic Exercise Capacity and Blood Pressure and Arterial Saturation During Exercise

A sub-maximal, self-paced, 6-min stepper test (6MST) was performed. Participants were all given the same instructions for the 6MST: to start at a pace they felt they could continue at for 6 min with the objective of completing as many steps as possible within 6 min. Full details are presented elsewhere. (Jones et al., 2017). This has previously been validated in this age-group against walking pace and sub-maximal oxygen consumption achieved in the 6 min walk test. (Jones et al., 2017). A portable expired gas analysis system including a Polar heart rate monitor (K4B2; COSMED, Rome, Italy) was used to measure breath-by-breath whole-body oxygen consumption (V̇O_2_) and heart rate during the 6MST. Exercise capacity was defined using 2 variables: 1) the number of steps achieved during the test and 2) the highest V̇O_2_ of a rolling 60 s average during exercise. The workload achieved during the test was estimated in Watts using methods previously described. (Jones et al., 2017).

Systolic and diastolic blood pressure (SBP and DBP) were measured during exercise using a specialist motion-tolerant blood pressure monitor (Tango M2 Stress Test Monitor, SunTech Medical, United States). Measurements were made one minute into the exercise test (during the second minute of exercise). Mean arterial pressure was calculated as *DBP+⅓(SBP-DBP)*.

Arterial saturation was measured from the finger throughout rest, exercise and recovery using a basic battery-powered clip-on pulse oximeter. This was checked periodically by the operator and the lowest value observed was recorded.

### Near-Infrared Spectroscopy Measurements

For both exercise and the arterial occlusion, a NIRS device (Portamon, Artinis Medical Systems, Netherlands) (Grassi and Quaresima, 2016; Jones et al., 2016) was positioned on the lateral head of the gastrocnemius where the calf girth was greatest, orientation was standardized, the device was secured and covered completely using a neoprene sleeve. TSI was estimated using spatially solved spectroscopy. The device was set to sample at 10 Hz. The 6MST was performed as specified above. During recovery a rapid inflation cuff (Hokanson, SC10D/E20; Washington, United States) was placed on the thigh proximal to the location of NIRS measurement. A protocol of short transient arterial occlusions was performed for 3 min immediately following the exercise test (data presented elsewhere). (Jones et al., 2021b). The staff member conducting the measurement then observed the NIRS signals until the participant was fully recovered and a steady-state had been reached before the cuff was inflated to a supra-systolic pressure (>250 mmHg) to induce complete arterial occlusion for at least 2 min.

Analysis of NIRS data was conducted using custom written programs in MATLAB R2014a (MathWorks Inc.). TSI was averaged over 5 s at each measurement time point: at rest, at the peak of the initial increment at onset of exercise, at the minimum observed during exercise and at the end of exercise. (Boezeman et al., 2016). The difference between TSI at rest (baseline) and at the initial increment (∆TSI_BL-INC_) was calculated as the increment minus the baseline value. The TSI change from baseline to the end of exercise (∆TSI_BL-EE_) was calculated as the baseline value minus the value at the end of exercise. The TSI change from the increment to the minimum ∆TSI_INC-MIN_ was calculated as the increment value minus the minimum value. All values were presented as positive despite ∆TSI_BL-EE_ being a negative change.

**FIGURE 1:**
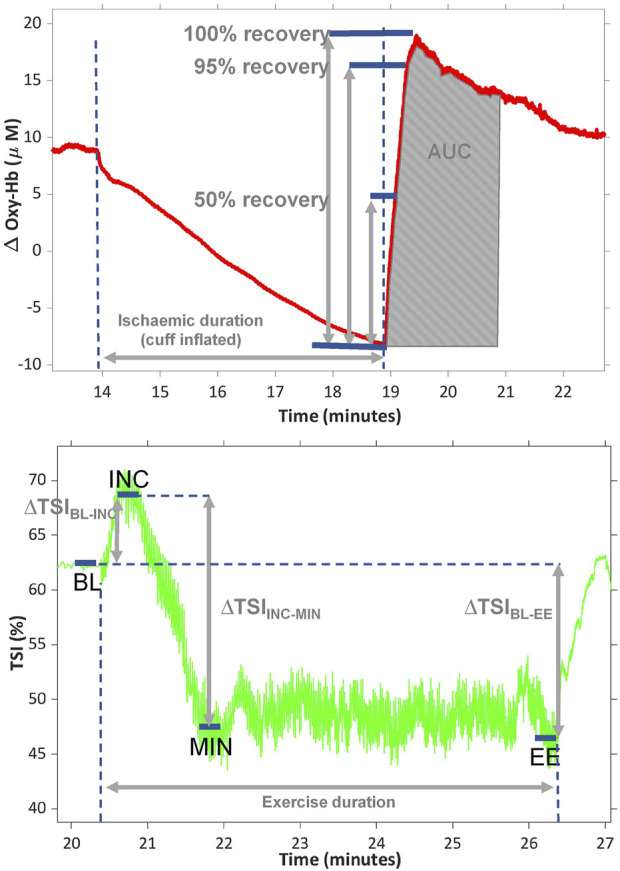
representative traces showing the change in oxygenated haemoglobin (oxy-Hb) during ischaemia and post occlusive reactive hyperaemia (PORH) (top) and change in tissue saturation index (TSI%) during exercise (bottom). Area under the curve (AUC) was calculated after normalising the start of PORH to zero. TSI was averaged over 5 s at baseline (BL), at the peak of the initial increment at onset of exercise (INC), at the minimum observed during exercise (MIN) and at the end of exercise (EE). ∆TSI_BL-INC_ is the difference between TSI at BL and INC; ∆TSI_BL-EE_ is the TSI change from BL-EE; ∆TSI_INC-MIN_ is the TSI change from INC to MIN. All values are presented as positive values, despite ∆TSI_BL-EE_ being a negative change.

Time to 50%, 95% and 100% peak PORH and area under the curve (AUC) for the PORH were calculated by following previously presented methods. (Bopp et al., 2014; Willingham et al., 2016). In short, all traces were inspected by eye, the point at which the cuff was released was identified from the oxy-Hb trace as the moment the first upward inflection point could be visualized and the peak response was identified as the maximum value captured within 2 min from cuff release. The change in oxy-Hb from the start to the end of the PORH (∆OxyHb) was calculated as the peak value minus the start value (Figure 1). Because the response to ischaemia is time-sensitive, we used single values rather than a 5-s average. The recovery rate was calculated as 1 divided by the time to 100% peak PORH. The oxy-Hb and TSI signals were not smoothed prior to post-processing analysis and all analysis was done by a single observer.

### Statistical Methods

Statistical analysis was carried out in STATA 17 (StataCorp College Station, TX, United States). Categorical data are presented as frequency (%). Continuous data were examined for normality; normally distributed sample data are summarised as means ± SD and skewed data as medians (interquartile range). Comparison of participant characteristics by sex was done using an unpaired Student’s *t* test for normally distributed continuous data, χ^2^ test for categorical data and a Wilcoxon Rank Sum test for skewed data (ATT).

Associations between exposures and outcomes are presented as unadjusted (unadj.) and adjusted (adj.) β-coefficients. Model 1a (M1a) is adjusted for confounders: age, sex and ethnicity and for the estimated workload achieved during the stepping test as an auxiliary co-variate. Model 1b (M1b) is adjusted for age, sex, ethnicity and mean arterial pressure (MAP) change at the start of exercise to account for the change in driving pressure at initiation of exercise. Models where indices of PORH are included as exposures are additionally adjusted for height, as an auxiliary co-variate to account for differences in hydrostatic pressure between participants and for the duration of the ischaemic stimulus.

## Results

### Participant Characteristics

In total, 711 participants who attended the SABRE visit 3 clinic undertook the 6-min stepper test and, of these, 558 participants underwent NIRS measures of TSI during exercise (mean age±SD: 73 ± 7years, 59% male). It was not possible to fit the NIRS-device to the lower leg in 153 participants due to either tight fitting clothing, broken or sensitive skin or because the participant declined to have the device fitted. In a sub-set of 149 participants, a leg cuff was fitted to the upper leg and participants were able to tolerate a ≥2 min arterial occlusion.

Participant characteristics are provided in Table 1 stratified by men and women for all participants and for the PORH sub-group only. By design the SABRE study recruited more men than women. (Tillin et al., 2012). On average, women were younger, had a higher BMI and a thicker layer of adipose over the measurement site and achieved lower exercise capacity.

**TABLE 1:** Participant characteristics stratified by sex. Values are mean ± SD or median (inter-quartile range) for the whole study group and those who undertook an ischaemic occlusion. ATT, adipose tissue thickness at the NIRS measurement site, BMI, body mass index, CVD, cardiovascular disease, ethnicity is E, European; SA, South Asian ; AFC, African Caribbean or OT, Other; T2DM, type-2 diabetes mellitus.

Characteristic	Mean ± SD or median (IQR) or *n(%)*					
	All (*n* = 558)			Sub-group (*n* = 149)		
	Women (*n* = 231)	Men (*n* = 327)	*p*	Women (*n* = 42)	Men (*n* = 107)	*p*
Age (years)	71 ± 6	75 ± 6	*<0.001*	71 ± 5	75 ± 6	*<0.001*
Ethnicity, *n* (E, SA, AFC, OT)	92,54,80,5	156,117,54,0	*<0.001*	18,9,14,1	43,50,14,0	*0.003*
BMI (kg/m^2^)	28.3 ± 4.9	27.2 ± 3.6	*0.004*	26.1 ± 4.1	26.6 ± 3.1	*0.481*
ATT (cm) (*n = 492*)	0.90 (0.71, 1.2)	0.53 (0.41, 0.67)	*<0.001*	0.78 (0.62, 1.09)	0.51 (0.40, 0.62)	*<0.001*
T2DM *n* (%)	51 (22)	75 (23)	*0.057*	5 (12)	26 (24)	*0.094*
CVD *n* (%) (*n = 539*)	13 (6)	57 (18)	*<0.001*	4 (10)	22 (21)	*0.135*
Steps achieved	190 ± 73	227 ± 78	*<0.001*	216 ± 65	238 ± 76	*0.073*
VO_2_ achieved (ml/kg/min) (*n = 495*)	14.7 ± 3.8	17.1 ± 4.4	*<0.001*	16.8 ± 3.6	17.9 ± 4.2	*0.125*
Occlusion duration (s)	-	-	*-*	208 ± 77	211 ± 66	*0.830*
Arterial saturation <95% during exercise *n* (%) (*n = 446*)	16 (8.3)	15 (6.0)	*0.345*	3 (8.6)	4 (4.6)	*0.393*

### ∆TSI During 6MST Versus Post-Occlusive Reactive Hyperaemia Measurements

Greater changes in TSI from rest to the end of exercise (∆TSI_BL-EE_) indicate larger drops in TSI during exercise. A positive association was observed between the time to recovery during PORH and ∆TSI_BL-EE_, such that slower times to recovery (time to 95% and 100% PORH) were associated with larger drops in TSI (table 2). Coefficients were similar after adjusting for co-variates in model 1a (table 2). The overall recovery rate during PORH (1/time to 100% PORH) was negatively associated with ∆TSI_BL-EE_. AUC and ∆Oxy-Hb during PORH were positively associated with ∆TSI_BL-EE_. Adjustment for co-variates did not alter these associations (table 2).

**TABLE 2:** Associations between TSI, tissue saturation index; changes during exercise and indices of post-occlusive reactive hyperaemia (PORH); measured in the lower-limb skeletal muscle. ∆TSI_BL-EE_ is the change in tissue saturation index (TSI); between rest (baseline, BL) and the EE, end of exercise ; ∆TSI_INC-MIN_ is the change in TSI from it is highest point at the INC, increment; to it is MIN, minimum; ∆TSI_BL-INC_ is the change in TSI from the BL to the highest point (INC). unadj, Unadjusted; and adjusted β-coefficients, 95% confidence intervals and *p*-values are presented for each association. M1a, Model 1a; is adjusted for age, sex, ethnicity, the duration of the ischaemic stimulus and the estimated workload achieved during the stepping test. M1b, Model 1b; is adjusted for age, sex, ethnicity and the duration of the ischaemic stimulus. M2, Model 2; is adjusted for age, sex, ethnicity, mean arterial pressure (MAP); change at the start of exercise.

	Β-coefficient (95%CI) *p-value*						
	∆TSI_BL-EE_		∆TSI_INC-MIN_		∆TSI_BL-INC_		
NIRS measure of PORH	Unadj. (*n* = 149)	M1a (*n* = 149)	Unadj. (*n* = 149)	M1a (*n* = 149)	Unadj. (*n* = 129)	M1b (*n* = 129)	M2 (*n* = 129)
**Time to 50% PORH (s)**	0.20 (−0.09, 0.49) *0.169*	0.14 (−0.17, 0.44) *0.366*	0.06 (−0.25, 0.36) *0.720*	−0.01 (−0.33, 0.31) *0.935*	−0.25 (−0.42, 0.07) *0.006*	−0.19 (−0.37, 0.006) 0.043	−0.18 (−0.36, 0.005) *0.056*
**Time to 95% PORH (s)**	0.09 (0.008, 0.17) *0.031*	0.08 (−0.01, 0.17) *0.085*	0.02 (−0.07, 0.10) *0.683*	−0.003 (−0.09, 0.10) *0.945*	−0.09 (−0.13, 0.04) *0.001*	−0.07 (−0.12, 0.02) 0.009	−0.07 (−0.12, 0.02) *0.012*
**Time to 100% PORH (s)**	0.05 (0.01, 0.08) *0.009*	0.05 (0.01, 0.09) *0.012*	−0.002 (−0.04, 0.04) *0.937*	0.003 (−0.04, 0.04) *0.903*	−0.04 (−0.06, 0.02) *0.001*	−0.03 (−0.05, 0.004) 0.024	−0.03 (−0.05, 0.003) *0.028*
**Recovery rate PORH (1/s)**	−47 (−81, 13) *0.006*	−47 (−85, 9.9) *0.014*	−11 (−48, 25) *0.538*	−9.8 (−49.6, 29.9) *0.626*	20 (−0.88, 40.7) *0.060*	8.3 (−14, 31) 0.468	7.2 (−15.4, 29.9) *0.530*
**(log)AUC PORH (µM x s)**	1.6 (0.62, 2.6) *0.002*	1.7 (0.62, 2.8) *0.002*	1.5 (0.45, 2.5) *0.005*	1.6 (0.45, 2.7) *0.007*	0.68 (0.06, 1.3) *0.032*	0.80 (0.14, 1.5) 0.018	0.85 (0.18, 1.5) *0.013*
**∆OxyHb PORH (µM)**	0.05 (0.02, 0.10) *0.042*	0.04 (0.002, 0.108) *0.041*	0.08 (0.03, 0.13) *0.003*	0.08 (0.03, 0.13) *0.004*	0.06 (0.03, 0.09) *<0.001*	0.06 (0.02, 0.07) 0.001	0.05 (0.02, 0.09) *0.001*

PORH recovery times and rate were not associated with the change in TSI from the increment to the minimum value (∆TSI_INC-MIN_). AUC and ∆Oxy-Hb during PORH were positively associated with ∆TSI_INC-MIN_ and adjustment for co-variates did not alter these associations (table 2).

PORH recovery times (time to 50%, 95% and 100% PORH) were negatively associated with the change in TSI from rest to the initial increment (∆TSI_BL-INC_), these associations were similar even after adjustment for co-variates including the change in MAP at the start of exercise (table 2). The rate of PORH recovery was positively associated with ∆TSI_BL-INC,_ but this association was attenuated by over half after adjustment for co-variates. AUC and ∆Oxy-Hb during PORH were positively associated with ∆TSI_BL-INC_ (table 2).

### Exercise Capacity and Post-Occlusive Reactive Hyperaemia

PORH recovery times were negatively associated with steps and VO_2_ achieved during the 6MST. These associations were largely explained by co-variates age, sex and ethnicity (table 3). Recovery rate, AUC and ∆Oxy-Hb were all positively associated with steps and VO_2_ achieved during the 6MST. The associations between AUC and ∆Oxy-Hb with steps achieved were only partially explained by co-variates (Table 3).

**TABLE 3:** Associations between exercise capacity measures (steps achieved and measured V̇O_2_) and indices of post-occlusive reactive hyperaemia (PORH); or tissue saturation index (TSI); changes during exercise measured in the lower-limb. ∆TSI_BL-EE_ is the change in TSI, between rest (baseline, BL) and the end of exercise (EE); ∆TSI_INC-MIN_ is the change in TSI from its highest point at the INC, increment; to its MIN, minimum; ∆TSI_BL-INC_ is the change in TSI from the BL to the highest point (INC).unadj, Unadjusted; and adjusted β-coefficients, 95% confidence intervals and *p*-values are presented for each association. M1, Model 1; is adjusted for age, sex and ethnicity. Associations between PORH measures and exercise capacity were also adjusted for height and ischaemic duration in M1. *indicates models also adjusted for estimated workload (W).

	Exercise capacity measured β-coefficient (95%CI)			
NIRS measures	Steps achieved		VO_2_ achieved (ml/min)	
	Unadj	M1	Unadj	M1
	*n = 153*	*n = 153*	*n = 148*	*n = 148*
**Time to 50% PORH (s)**	−5.3 (−10.0,-0.66) *0.026*	−2.7 (−7.1.1.7) *0.221*	−21.9 (−44.8.1.0) *0.061*	−6.2 (−25.7.13.2) *0.528*
**Time to 95% PORH (s)**	−1.3 (−2.6.0.04) *0.056*	−0.42 (−1.7.0.87) *0.525*	−6.1 (−12.5.0.36) *0.064*	−0.54 (−6.3.5.2) *0.852*
**Time to 100% PORH (s)**	−0.29 (−0.86.0.28) *0.315*	0.11 (-0.48.0.70) *0.709*	−1.3 (−4.1.1.4) *0.339*	1.1 (−1.5.3.7) *0.406*
**Recovery rate PORH (1/s)**	699 (140,1259) *0.015*	306 (−235,848) *0.266*	3835 (1127,6542) *0.006*	1383 (−1016,3782) *0.256*
**AUC PORH (µM x s)**	20.8 (4.8.36.7) *0.011*	18.0 (2.3.33.7) *0.025*	90.8 (12.0,169.6) *0.024*	22.5 (−48.2.93.1) *0.531*
**∆OxyHb PORH (µM)**	1.3 (0.46.2.1) *0.002*	0.86 (0.10.1.6) *0.027*	4.4 (0.21.8.7) *0.040*	−0.13 (−3.8.3.5) *0.944*
	*n = 558*	*n = 558*	*n = 495*	*n = 495*
**∆TSI**_**BL-EE**_	4.1 (2.6.5.6)	1.7 (0.49.2.9)	24.7 (17.8.31.7)	7.7 (3.2.12.3)
	*<0.001*	0.006*	*<0.001*	0.001*
**∆TSI**_**INC-MIN**_	3.6 (1.9.5.2)	2.5 (1.3.3.8)	16.4 (8.7.24.1)	6.6 (1.9.11.3)
	*<0.001*	<0.001*	*<0.001*	0.006*
	*n = 478*	*n = 478*	*n = 427*	*n = 427*
**∆TSI**_**BL-INC**_	2.8 (0.14.5.4)	−1.2 (-3.6.1.3)	24.0 (11.8.36.1)	1.7 (-9.3.12.7)
	*0.039*	*0.352*	*<0.001*	*0.764*
**∆TSI**_**BL-INC**_	2.9 (0.22.5.5)	−1.1 (−3.5.1.3)	24.9 (13.0.36.9)	2.2 (−8.5.12.8)
**Adj. ∆MAP**	*0.034*	*0.381*	*<0.001*	*0.687*

∆TSI_BL-EE_ and ∆TSI_INC-MIN_ were positively associated with exercise capacity outcomes, these associations were only partially explained by co-variates age, sex and ethnicity (table 3). ∆TSI_BL-INC_ was positively associated with both exercise capacity outcomes, these associations were largely explained by co-variates age, sex and ethnicity, however, adjustment for ∆MAP at the start of exercise did not affect β-coefficients (table 3).

**FIGURE 2:**
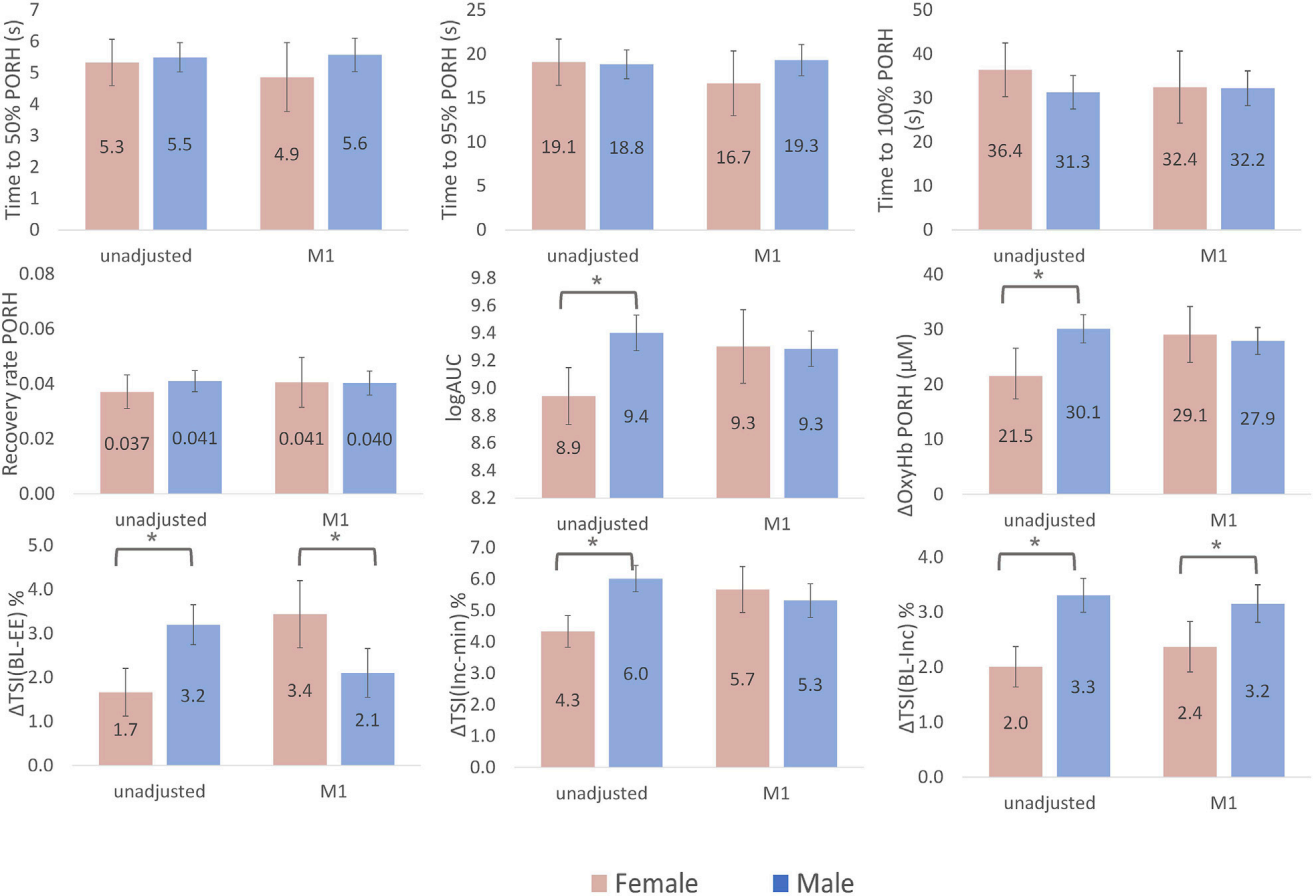
Sex differences in NIRS-measured skeletal muscle microvascular response to cuff-induced ischaemia and exercise. Bars represent marginal means for female (pink) and male (blue). Error bars are 95% confidence intervals. ∆TSI_BL-EE_ is the change in tissue saturation index (TSI) between rest (baseline, BL) and the end of exercise (EE); ∆TSI_INC-MIN_ is the change in TSI from it is highest point at the increment (INC) to it is minimum (MIN); ∆TSI_BL-INC_ is the change in TSI from the BL to the highest point (INC). Area under the curve (AUC) had a skewed distribution and so log transformed values are presented. Model 1 (M1) is adjusted for age, ethnicity, adipose tissue thickness (ATT), height, ischaemic duration and presence of type 2 Diabetes and cardiovascular disease. The change in tissue saturation index (∆TSI) from baseline to end of exercise (BL-EE) and ∆TSI from increment to minimum (Inc-min) were additionally adjusted for workload achieved during exercise and the ∆TSI from baseline to the initial increment (BL-Inc) was additionally adjusted for change in mean arterial pressure at the beginning of exercise in model 1.

Time to 50%, 95% and 100% recovery during PORH and the overall recovery rate were not different in men versus women (Figure 2). The AUC and ∆oxy-Hb were smaller in women versus men, however, both differences were explained by adjustment for co-variates (age, ethnicity, ATT, T2DM and CVD). ∆TSI_BL-EE_ was 1.5% greater in men versus women, however, after adjustment for co-variates (including estimated workload achieved during stepping), this difference was reversed to a 1.3% greater change in women (Figure 2). A similar pattern was observed for ∆TSI_INC-MIN_ but values were similar between men and women after adjustment. ∆TSI_BL-INC_ was 1.3% greater in men versus women, this difference persisted after adjusting for co-variates (Figure 2).

## Discussion

This study presents evidence that improved microvascular reactivity in response to ischaemia is associated with greater increments in TSI pattern at initiation of exercise and smaller overall drops in TSI during exercise. In addition, we show that these local microvascular measures are associated with exercise capacity in older adults and that differences between men and women enrolled in a population-based cohort can be detected using NIRS.

### Exercise Deoxygenation and Post-Occlusive Reactive Hyperaemia

For the first time, we describe a relationship between greater skeletal muscle deoxygenation from the start to the end of exercise (greater ∆TSI_BL-EE_ values) and blunted (slower) PORH recovery rates. Previously, greater deoxygenation during exercise has been described in various conditions of vascular dysfunction, (Bauer et al., 2004; Boezeman et al., 2016) including PAD (Bauer et al., 2004; Vardi and Nini, 2008; Mesquita et al., 2013) and chronic lower limb compartment syndrome. (van den Brand et al., 2005). A blunted reactive hyperaemia, irrespective of measurement method, is widely accepted as a measure of impaired vascular function (Rosenberry and Nelson, 2020) and predictor of future cardiovascular events. (Anderson et al., 2011). NIRS has previously been applied to assessed PORH and measures found to be impaired in patients with PAD, (Kragelj et al., 2001) various other clinical populations (Doerschug et al., 2007; Mayeur et al., 2011) and in old, compared to young, adults. (Rosenberry et al., 2018). As both response to exercise and PORH response measures are thought to represent a haemodynamic insufficiency, our finding that these measures are associated was expected. We did not observe statistically significant associations between ∆TSI_BL-EE_ and time to 50% PORH and the association between ∆TSI_BL-EE_ and time to 95% PORH was attenuated after adjustment for co-variates. This could be due to different physiological components underpinning the initial versus the final stages of the PORH response curve. However, these associations were positive and the β-coefficient for time to 95% PORH was only marginally altered after adjustment which is in line with the robust associations we observed between ∆TSI_BL-EE_ and time to 100% PORH, recovery rate, AUC and ∆oxy-Hb. Further work is necessary to establish if the initial fast PORH response versus the slow response differ in their relationship with skeletal muscle deoxygenation during exercise.

Conversely, the TSI change from its highest point (the initial increment) to its lowest (∆TSI_INC-MIN_) was only associated with AUC and ∆OxyHb derived from PORH. The lack of association between ∆TSI_INC-MIN_ and the PORH recovery rate and times, could be due to this parameter also encompassing the change in TSI at initiation of exercise (∆TSI_BL-INC_) which may have amplified ∆TSI_INC-MIN_ if there were favourable vasodilatory response at initiation of exercise.

We speculate that larger ∆TSI_BL-INC_ values indicate better peripheral vasodilatory capacity at exercise onset. We observed strong associations between the change in TSI at initiation of exercise and PORH measures. Larger values of ∆TSI_BL-INC_ were associated with faster PORH recovery times suggesting this novel parameter is closely related to an appropriate haemodynamic response to demand for oxygenated blood. Adjustment for the change in MAP at the start of exercise, to account for the increased driving pressure, did not alter associations, it is therefore likely that greater values of ∆TSI_BL-INC_ indicate adequate perfusion, or perhaps over-perfusion, in response to the increase in metabolic demand within skeletal muscle at the onset of exercise. As this was a sub-maximal, self-paced exercise test, it is unlikely that participants would have initiated exercise at a workload near to their maximum, therefore maximum circulatory capacity would not have been achieved and over-perfusion would be possible. Future research is necessary to verify these findings and ∆TSI_BL-INC_ merits investigation in clinical studies in order to determine threshold values that could inform on peripheral haemodynamic insufficiency.

### Exercise Capacity

Blunted skeletal muscle oxygen kinetics during PORH were associated with poorer exercise capacity, both in terms of steps completed and VO_2_ achieved. Adjustment for co-variates, age, sex and ethnicity attenuated these estimates towards the null which is expected given the known decline in microvascular function with age. However, the associations between AUC, ∆OxyHb and steps remained statistically significant after adjustment for co-variates. This is in line with previous findings linking microvascular reactivity and physical activity levels. (Montero et al., 2015; Lanting et al., 2017). However, this association has not previously been described using NIRS-measured PORH. Previous studies typically investigate down-stream PORH using venous occlusion plethysmography or laser-Doppler. While these techniques inform us on 1) the overall blood influx to the whole of the lower limb (plethysmography) and 2) the response within the cutaneous circulation (laser Doppler), NIRS provides assessment of changes specifically in microvasculature and, typically, a large portion of the signal is from skeletal muscle. Therefore, our findings highlight the importance of developing complementary methods of assessing skeletal muscle.

Deoxygenation across exercise (∆TSI_BL-EE_) was positively associated with exercise capacity measures. However, the use of ∆TSI_BL-EE_ to assess microvascular function in the context of the self-paced exercise test carried out here is limited because this association is heavily confounded by the effect of the achieved workload during exercise. After adjustment for estimated workload, effect sizes were attenuated by half for steps completed and nearly 3 times for VO_2_ achieved. The change in TSI at exercise onset, ∆TSI_BL-INC_, is likely to be less dependent of workload achieved and has not previously been explored in the context of microvascular function and exercise capacity. These results show positive associations between ∆TSI_BL-INC_ and both exercise capacity outcomes suggesting that improved vaso-dilatory capacity in the microvasculature of skeletal muscle is associated with improved exercise capacity. Adjustment for ∆MAP at the start of exercise did not affect β-coefficients.

### Sex Differences in Microvascular Reactive Hyperaemia in Older Adults

During PORH, only AUC and ∆oxy-Hb differed by sex, with smaller values in women suggesting reduced microvascular reactivity. Although the time to 100% recovery and the recovery rate were slightly faster in men, these differences did not reach statistical significance. Our findings are in line with two previous studies that detected more rapid and greater magnitude of downstream reactive hyperaemia in men versus women using NIRS. (Fellahi et al., 2014; Rasica et al., 2022). These studies enrolled young healthy individuals and were unable to adjust for confounding factors, such as ATT, due to small sample sizes. The differences presented here were largely explained by adjustment for co-variates (age, ethnicity, ATT, T2DM and CVD). Studies investigating sex-differences in the upstream response to ischaemia, examined using flow-mediated dilatation (FMD), have yielded conflicting results. (Levenson et al., 2001). Inconsistencies are thought to be related to confounding from the differences in vessel diameter at rest by sex. This further highlights the importance of developing additional complementary techniques that directly assess downstream reactive hyperaemia.

Deoxygenation during exercise was lower in women when differences were unadjusted, however, after adjustment (Model 1) deoxygenation was greater in women. Adjusting for the workload achieved during exercise and ATT contributed to this reversal of effect as men achieved a higher estimated workload during exercise (a contributor towards greater deoxygenation) and women had greater ATT. Greater ATT can attenuate the signal from skeletal muscle resulting the appearance of less deoxygenation. The ∆TSI_BL-INC_ was greater in men than women despite adjustment for all co-variates. Together this pattern of tissue oxygenation change during exercise suggests that, in older adults, women have a reduced vasodilatory capacity at the start of exercise and haemodynamic insufficiency at the microvascular level throughout exercise. This finding is in line with previously described sex differences in the pattern of cardiovascular aging at the population level. (Redfield et al., 2005; Chung et al., 2006). The higher prevalence of heart failure with preserved ejection fraction in ageing women versus men is thought to be underpinned by, among other things, greater microvascular dysfunction. (Beale et al., 2018). Early detection of dysfunction within skeletal muscle could lead to a more targeted intervention to mitigate risk in women.

### Study Limitations

We used a self-paced exercise test to measure exercise capacity in our older adult population. As workload was not controlled, we cannot be certain that changes in TSI measured during exercise were not due to self-selected higher workloads and we cannot be sure participants did not change the rate they were stepping at throughout the test. However, we endeavoured to address this via adjustment of statistical models for an estimate of the workload achieved. Furthermore, activities of daily living rarely occur at peak exercise capacity, therefore, a self-paced exercise stimulus may provide more appropriate insight into daily exercise limitations in older adults compared to traditional graded exercise tests. The occlusion duration used to apply ischaemic changes was variable between participants. We targeted a 5-min duration in all participants but found that this duration was poorly tolerated in our study population. We report ischemic duration in table one and show no difference between men and women, we also adjusted statistical models for the duration of the ischaemic stimulus. Furthermore, previous work suggests that above a threshold stimulus of 1.5 min of cuff occlusion there is an association between flow and vessel dilatation as measured by FMD. (Leeson et al., 1997). we therefore selected an arterial occlusion >2 min as our threshold for inclusion in results and present an average tolerated duration of ∼3.5 min which is well above this threshold.

## Conclusion

This study suggests that NIRS-derived PORH parameters and the pattern of TSI change measured during exercise offer a simple approach to evaluation of microvascular function. Furthermore, these methods are both sensitive enough to detect sex differences in the skeletal muscle microvasculature of ageing populations. Developing methods that capture local changes from skeletal muscle is an important step towards understanding the reduction in exercise capacity with age and detection of early, pre-clinical changes which may lead to onset of cardiovascular disease.

## Data Availability

The raw data supporting the conclusions of this article will be made available by the authors, without undue reservation.
